# Functional Linking Between Negative and Positive ERPs for Syntactic Processing in Japanese: Mutual Enhancement, Syntactic Prediction, and Working Memory Constraints

**DOI:** 10.3389/fpsyg.2019.02744

**Published:** 2019-12-13

**Authors:** Shingo Tokimoto, Yayoi Miyaoka, Naoko Tokimoto

**Affiliations:** ^1^Department of English Language Studies, Mejiro University, Tokyo, Japan; ^2^Department of Liberal Arts, Hiroshima University of Economics, Hiroshima, Japan; ^3^Department of Life Management, Shobi University, Saitama, Japan

**Keywords:** P600, N400, event-related spectral perturbation, intertrial phase coherence, Japanese Reading Span Test, syntactic prediction, syntactic island, head direction

## Abstract

This study attempts to detect the differences in event-related potentials (ERPs) associated with two syntactic processes: the syntactic integration of discontinuous dependency and the detection of a violation of the syntactic island constraint. We recorded the electroencephalogram elicited by complex sentences in Japanese that included a dependency between a quantifier and its head noun, in which we changed the word order of the two words to manipulate the presence and absence of a syntactic integration and a syntactic island violation while keeping the lexical items and construction unchanged. We found significant negative and positive deflections for the syntactic integration only when a quantifier preceded its head noun. We also observed significant negative and positive deflections for the syntactic island violation, for which the negativity was more salient when a quantifier preceded its head noun. This study is the first to report a late positive ERP for a violation of the syntactic island constraints in Japanese, and the results showed that the ERP elicited by syntactic integration and that by syntactic island violation were different in terms of their latency, topography, and duration. More importantly, the ERPs elicited by the two syntactic processes were biphasic, and the amplitudes of the negative ERP and of the positive ERP were positively correlated. This positive correlation could be a characteristic of syntactic processing because it contrasted with the negative correlation reported for the ERP elicited by semantic anomalies in English. Furthermore, the amplitude of the ERP for syntactic integration was negatively correlated with the individual capacity of working memory (WM). That is, a reader with greater WM capacity showed smaller negativity and positivity for the syntactic integration, whereas the amplitude for the syntactic island violation showed no significant correlation with the individual capacity of WM. Our results suggested that linguistic ERPs functionally interacted with each other and that the ERP involving the retention and the retrieval of a distant word could be constrained by the individual differences in WM capacity. We discuss the possible reasons for the contrast between English and Japanese on the basis of the cross-linguistic differences in the two languages.

## 1. Introduction: Causes of Linguistic Late Positive ERPs

The study of linguistic event-related potentials (ERPs) has contributed much to examining the structure of linguistic knowledge and the mechanism of linguistic processing. The ERPs that have been most frequently discussed are a negative ERP that peaks at approximately 400 ms after the onset of a stimulus (N400) and a positive ERP with a peak latency at approximately 600 ms (P600). Kutas and Hillyard (1980) is known as the pioneering research on the N400. Kutas and Hillyard (1980) visually presented the sentences in (1) word by word with the stimulus onset asynchrony (SOA) set to 1 s and observed the N400 widely across the scalp (i.e., at the Fz, Cz, and Pz locations) after the presentation of *socks* in (1-b) against *work* in (1-a). On the other hand, the capitalized *SHOES* in (1-c) elicited a positive ERP with a peak at 560 ms.


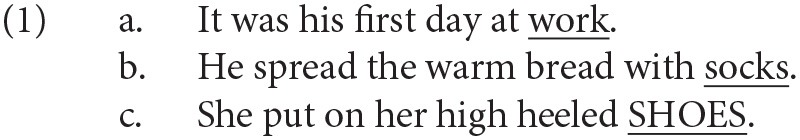


Kutas and Hillyard (1980) proposed that the N400 was elicited by semantically anomalous information, and since then, the N400 has been understood as a manifestation of semantic processing.

Neville et al. (1991) is among the earliest studies on the P600. Neville et al. (1991) visually presented the sentences in (2) word by word with the SOA set as 500 ms and the ISI set as 200 ms, and they observed a positive ERP component for *of* underlined in (2-b) against (2-a) mainly in the occipital region in the time window of approximately 500–700 ms.


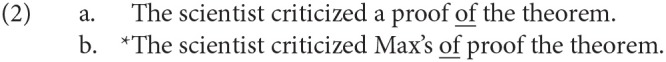


The positive ERP in (2-b) was understood as a manifestation of a syntactic constraint violation because *of* in (2-b) violates the phrase structure rules. The P600 has generally been interpreted as an indication of syntactic processing.

However, many researchers have reported several linguistic phenomena for which the P600 was observed, and it is now difficult to uniquely specify the underlying processes of the P600. This study tries to differentiate the linguistic late ERPs elicited by two syntactic phenomena in Japanese, namely, the syntactic integration of discontinuous dependency and the violation of syntactic island constraints, with the same lexical items and in the same construction for a better understanding of linguistic ERPs. In doing so, we will analyze the correlations between the positive ERP, the co-occurring negative ERP, and the individual capacity of working memory (WM). Furthermore, we will perform a time-frequency analysis and analyze the event-related spectral perturbation (ERSP) and the intertrial phase coherence (ITC) to examine the neural substrates of linguistic ERPs in detail.

We will begin with a brief survey of the linguistic phenomena that elicit the P600, and we will describe the background and the objectives of this study. The linguistic phenomena that are known to elicit the P600 are syntactic integration, syntactic constraint violation, syntactic reanalysis, and semantic anomalies.

### 1.1. Syntactic Integration

In a sentence in a natural language, two words that are discontinuous in time can have a stronger semantic relationship than their adjacent words. One of the common examples is English interrogative sentences as in (3), in which the sentence-initial *wh*-word has a stronger relationship with the verb *want* than the intervening *do* and *you*.


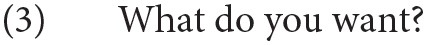


In (4-b), *which pop star* at the beginning of the subordinate clause functions as the object of the subordinate verb *imitated*, and therefore, the two are assumed to be syntactically integrated for their interpretation in real time. In (4-a), on the other hand, we find no syntactic integration of discontinuous constituents. Kaan et al. (2000) visually presented (4) word by word to participants with the SOA and the ISI set to 500 and 200 ms, respectively, and they observed a positive ERP for *imitated*, mainly in the occipital region, with a time window of 500–700 ms. This positive ERP was understood as a manifestation of syntactic integration.


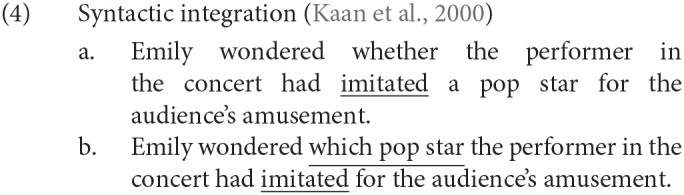


### 1.2. Violation of Syntactic Island Constraints

In (5-a), the *wh*-phrase *which of his staff members* is placed at the beginning of the subordinate clause in a similar way to (4-b), but it cannot be interpreted as the object of the sentence-final particle *by*. Here, the adverbial clause *when his son was questioned by (which of his staff members)* blocks the discontinuous dependency between *which of his staff members* and *by*. A constituent that blocks a discontinuous dependency crossing its boundary is called a “syntactic island” in the linguistic literature. The adverbial clause in (5-a) is an example of a syntactic island, and the discontinuous dependency between *which of his staff members* and *by* is assumed to violate the syntactic island constraint. In (5-b), on the other hand, no discontinuous dependency is involved. McKinnon and Osterhout (1996) visually presented (5) word by word with the SOA and ISI set to 400 and 100 ms, respectively, and they observed the P600 for *when* in (5-a) against (5-b) in the centro-parietal and occipital regions. The P600 was understood as a manifestation of a syntactic island constraint violation.


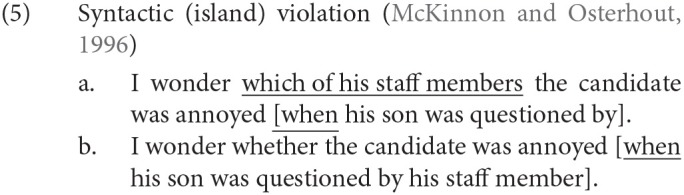


### 1.3. Syntactic Reanalysis

The third linguistic phenomenon that elicits the P600 is syntactic reanalysis. *The defendant* in (6-a) is temporarily ambiguous between the matrix object and the subordinate subject, and it is preferably interpreted as the former. *The defendant* is thus reanalyzed as the subordinate subject for the input of *was*. Osterhout et al. (1994) visually presented (6) word by word with the SOA and the ISI set to 650 and 300 ms, respectively, and they observed a positive ERP for *was* in (6-a) against (6-b) with the time window of 500–800 ms in the occipito-parietal region.


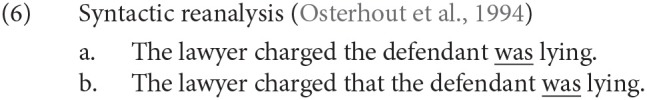


The P600 was understood as a manifestation of syntactic reanalysis.

### 1.4. Semantic Anomaly

A semantic anomaly was the last linguistic phenomenon that has been reported to elicit the P600. *Eat* in (7-b) is placed where a verb can appear, and therefore, we find no syntactic violation in (7-b) when we assume syntactic constraints to be the combinations of parts of speech. However, Kuperberg et al. (2003) visually presented (7) word by word with the SOA set as 400 ms and the ISI set as 100 ms, and they observed a positive ERP for *eat* in (7-b) against (7-a) with the time window of 500–800 ms. The positive ERP in (7-b) was understood to be elicited by the semantic anomaly.


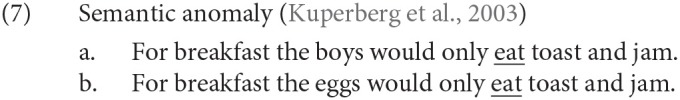


The P600 was long understood to be a manifestation of syntactic processing, whereas the N400 was associated with semantic processing. Therefore, after the finding that the P600 was elicited by a semantic anomaly, researchers have re-examined the sources of the P600, and the processing behind the P600 is still under intense discussion (Gouvea et al., 2010; Brouwer et al., 2012; Brouwer and Crocker, 2017). However, the exact comparison between each finding is difficult because the experiments that reported the P600 are different in terms of the constructions, languages, and experimental settings.

To examine the processing behind the P600 with the possibility that different lexical items and constructions can change the latency, the amplitude, and the duration of an ERP, Gouvea et al. (2010) recorded the electroencephalogram (EEG) elicited by English sentences, in which the lexical items and the constructions were controlled to be as similar as possible in different experimental conditions. The sentences in (8) are examples of the experimental sentences from Gouvea et al. (2010), and they analyzed the ERP at *show(ed)*.


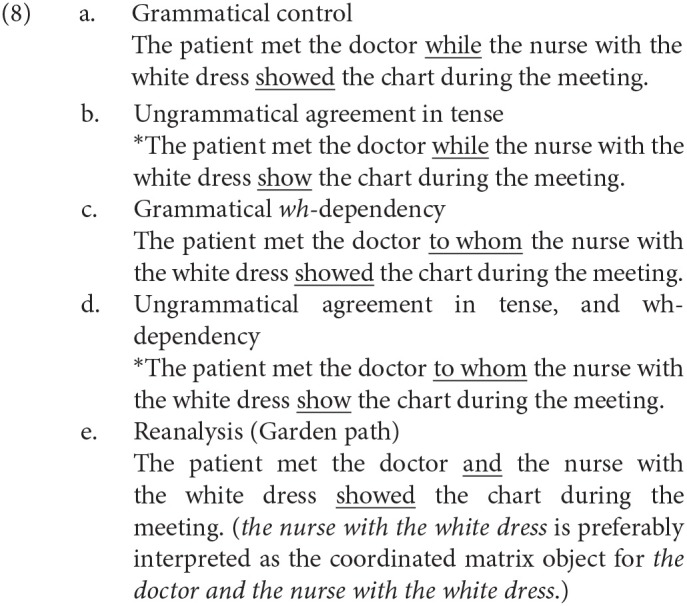


As a result, Gouvea et al. (2010) observed a parieto-occipital positive ERP with the time window of 500–1,100 ms for (8-b-e). However, the parietal positivity for *wh*-dependency in (8-c) was smaller in amplitude than those for (8-b,e), and another positive ERP was observed for (8-c) in the frontal region with the time window of 300–500 ms, which was more salient than the parietal positive ERP. Gouvea et al. (2010) did not discuss the ERP elicited by the violation of a syntactic island constraint.

Theoretical linguistics has a long tradition of assuming modularity for language faculty, and the linguistic ERPs are often interpreted as the evidence for such modularity. This is because their polarity, latency, and topography often differed depending on the kind of linguistic phenomenon. After the finding of “semantic P600,” however, researchers are required to be careful in directly corresponding the categories of the well-known ERP components (early left anterior negativity (ELAN), left anterior negativity (LAN), N400, P600, and so forth) to linguistic phenomena or constraints. We believe that researchers are expected to reinterpret the ERP components.

Note that several studies have observed the mutual dependency between the N400 and the P600 (Hoeks et al., 2004; van Herten et al., 2006; Van Petten and Luka, 2006; van de Meerendonk et al., 2008; Kim and Sikos, 2011); van de Meerendonk et al. (2008) is one of the earliest studies among them. van de Meerendonk et al. (2008) changed one word in the Dutch sentences in (9) to manipulate their plausibility to examine the effect of the degree of a semantic anomaly on the ERP. The sentence in (9-a) is one of the plausible grammatical sentences, and (9-b,c) are made to be semantically anomalous by replacing one word in (9-a). That is, *netvlies* (retina) in (9-a) is replaced by *wenkbrauw* (eyebrow) in (9-b) and is replaced by *sticker* (sticker) in (9-c). The N400 was observed for *wenkbrauw* in (9-b), and the N400 and the P600 were observed for *sticker* in (9-c) against *netvlies* in (9-a). According to the monitoring hypothesis proposed by van de Meerendonk et al. (2008), the P600 is a manifestation of the reanalysis to examine the possibility of a misanalysis by the monitoring mechanism.


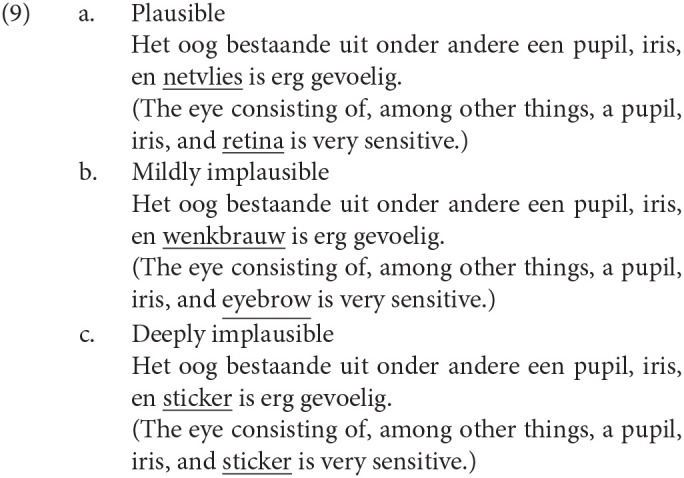


According to van de Meerendonk et al. (2008), a sentence with a serious semantic anomaly elicited the P600, and the N400 and the P600 were not the manifestations of the different kinds of linguistic constraints. van de Meerendonk et al. (2008) also claimed that the P600 counteracted the N400.

More recently, Kim et al. (2018) visually presented the semantically anomalous sentences in (10) word by word and observed the N400 and the P600 for *devouring* in (10-b,c) against *devoured* in (10-a). “Semantic attraction” in (10-b) means that the sentence can be appropriately interpreted by the reanalysis replacing *devouring* with *devoured* (Kim and Osterhout, 2005). On the other hand, (10-c) cannot be a plausible sentence by replacing *devouring* with *devoured*, even though we find no violation in the series of parts of speech.


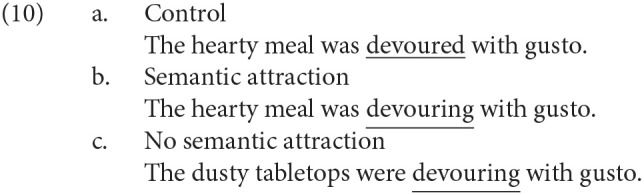


The amplitudes of the N400 and of the P600 in (10) were significantly negatively correlated with each other. That is, a reader that showed a greater N400 showed a smaller P600. Furthermore, the verbal WM capacity of a participant evaluated by the reading span task (Daneman and Carpenter, 1980) and the keep-track task (Miyake et al., 2000) was positively correlated with the amplitude of the P600 and negatively correlated with that of the N400. Kim et al. (2018) suggested that the N400 and the P600 effects were functionally linked in a trade-off relationship, constrained by individual differences in cognitive ability. In the current study, we attempted to examine several linguistic ERPs in detail, paying special attention to the relationships between the ERP components and between the ERP amplitude and the individual WM capacity.

We have reviewed four linguistic phenomena that were reported to elicit the P600, more precisely a late positive ERP. Note that the word order can be relevant to the neural activity in syntactic integration. Syntactic integration in English is often discussed as “filler-gap dependency” because a *wh*-phrase at the beginning of a sentence or a subordinate clause functions as the filler for the gap assumed after the corresponding word. Therefore, the input of the word corresponding to a *wh*-phrase can be predicted at the *wh*-phrase, and thus the lexical information of the *wh*-phrase and its prediction are assumed to be retained in WM until syntactic integration is established. Thus, the constraints of WM will be involved in syntactic integration in English. However, it is not cross-linguistically true in the syntactic integration of discontinuous constituents that the following word is always predicted at the input of the preceding phrase. That is, we can find an example of syntactic integration for which the syntactic prediction of the following word is absent. A quantifier in Japanese can be displaced from its head noun since the word order is relatively free in the language. The displacement of a quantifier from its head noun is called “quantifier floating” in the linguistic literature. The sentences in (11) are two examples of quantifier floating. The propositional meaning is the same in (11-a,b), and a quantifier *roku-mai* (six) precedes its head noun *shashin* (picture) in (11-a), whereas the head noun precedes the quantifier in (11-b)^1^.


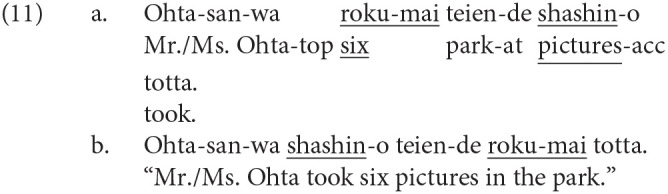


A syntactic integration process of *roku-mai* and *shashin* is expected at *shashin* in (11-a) and *roku-mai* in (11-b). In (11-a), the following input of the head noun can be predicted at the quantifier, whereas at *shashin* in (11-b), a syntactic integration with a quantifier is not predicted. We can thus examine the effect of syntactic prediction on the syntactic integration process by changing the order of the quantifier and its head noun as in (11-a,b)^2^.

Furthermore, we can manipulate the possible violation of a syntactic island constraint by placing a quantifier or the head noun in a potential syntactic island with the same lexical items and keeping the (possible) propositional meaning unchanged as in (12). The constituent indicated by square brackets *teien-o sanpo-shitsutsu* (park-acc a walk-taking) in (12) can function as a syntactic island of the adverbial clause, and therefore, the dependency construction between the quantifier (*roku-mai*) and the head noun (*shashin*) can be difficult in (12-b,d) because one of the two is placed in the possible island. The subordinate subject of the adverbial clause in (12) is understood as *Ohta-san* (Mr./Ms. Ohta) because a constituent can be phonetically null in Japanese when the referent is specified by the context.


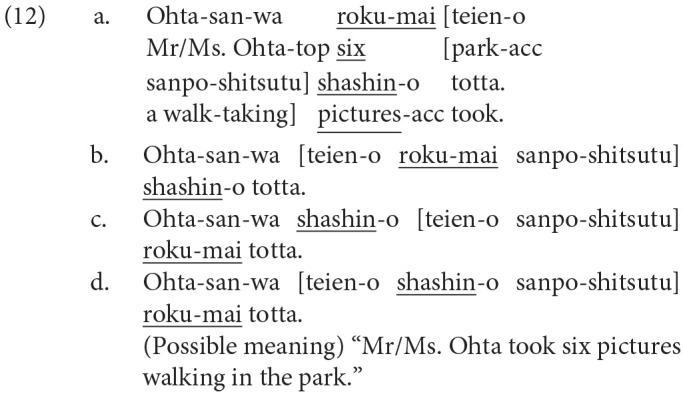


In the current study, we changed the word order of a complex sentence, including the dependency between a quantifier and its head noun as in (12), to manipulate (a) the presence or absence of a syntactic integration of discontinuous constituents, (b) the presence or absence of a syntactic prediction of the corresponding word (head noun), and (c) the presence or absence of the (possible) violation of a syntactic island constraint. In this way, we will try to differentiate the possible late positive ERPs elicited by a syntactic integration that can be modified by the syntactic prediction from those elicited by the violation of a syntactic island constraint. The close examination of linguistic ERPs in Japanese can have a cross-linguistic significance in the discussion of the universality and the peculiarity of language processing.

We will also discuss ERSP to characterize the time frequency properties of the ERPs expected for the syntactic integration and the violation of a syntactic island constraint. This is because ERPs can differ in their frequency properties even when we find no significant difference in their waveforms. As one of the studies that examined ERSP in language processing, Maguire et al. (2010) recorded EEGs of twenty healthy adults that listened to thematically related (e.g., leash-dog), taxonomically related (e.g., horse-dog), or unrelated (e.g., desk-dog) noun pairs. Maguire et al. (2010) observed a significant difference in ERSP between thematically and taxonomically related word pairs, whereas they observed no significant difference between them in the ERP. According to Maguire et al. (2010), the θ power increased over right frontal areas for thematic vs. taxonomic relationships, and the α power increased over parietal areas for taxonomic vs. thematic relationships. More recently, Schneider and Maguire (2018) examined the relationship between an ERP and the time frequency analysis by a syntactic and a semantic anomaly in English. Schneider and Maguire (2018) demonstrated that the N400 was associated with the power increase in the θ band, whereas the P600 was associated with the suppression in the β band. We could thus expect a difference in ERSP for the syntactic integration and the syntactic island violation. Furthermore, we will discuss the ITC associated with the two processes to distinguish between their evoked and induced neural activities.

One of the main reasons why we paid special attention to the syntactic integration and the possible violation of a syntactic island constraint is the specificity of discontinuous dependency to human language. Discontinuous dependency indicates that the set of sentences of natural language exceeds the generative capacity of finite-state grammar. This phenomenon is thus important for studying the computational aspects of natural language, and it has been intensively discussed in theories of syntax and sentence processing. The island phenomenon is assumed to be cross-linguistic, although the syntactic categories that constitute islands can vary among languages (Goodluck and Rochemont, 1992). It can be theoretically important here that the island effect in Japanese is much weaker than in English (Tokimoto, 2019). Discontinuous dependency is universal, and therefore, a possible contrast between the syntactic integration and the island constraint violation in Japanese can be important in the discussion of the universality and the specificity in sentence processing. This is the background of our study to examine neural activities that are peculiar to Japanese discontinuous dependency.

We enumerate our research questions in (13).


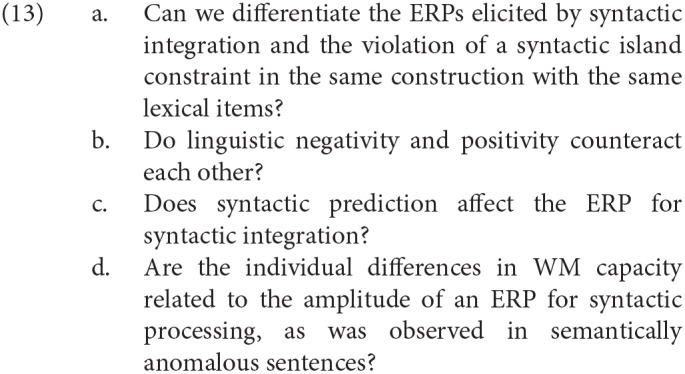


We will describe the methods used in our experiment in the next section.

## 2. Methods

### 2.1. Participants

Twenty-one native speakers of Japanese between 20 and 40 years old (*M* = 22.33 years, *SD* = 4.18 years, 6 men), participated in this study for payment. They were undergraduate students, graduate students, and company workers. The participants had normal or corrected-to-normal vision and had no history of neurological/psychiatric disorders. All the participants were right-handed, as assessed by the handedness questionnaire (Oldfield, 1971).

The individual capacity of WM was measured by the Japanese Reading Span Test (JRST) (Osaka, 2002). The Reading Span Test, originally developed by Daneman and Carpenter (1980), closely relates to language comprehension ability. A high score on this test is generally understood to be a manifestation of language processing efficiency (Daneman and Carpenter, 1980; Masson and Miller, 1983 for English and Osaka and Osaka, 1994 for Japanese). In the JRST used in this study, the participants read a set of unrelated sentences aloud on a computer screen without pausing between sentences. At the end of a set, they were asked to recall all the target words, which were underlined in red, in the sentences in the set. They were instructed not to state the target word in the last sentence of a set first. The participants were initially given five sets with two sentences per set, followed by five three-, five four-, and five five-sentence sets. The JRST included twenty-five sets of seventy sentences. Here, we adopted the number of trials in which the target words were correctly given as the index of the individuals' WM capacity. The index ranges from 0 to 70. The mean for our twenty-one participants was 41.40 (*SD* = 9.20). This study was approved by the ethics committee of Mejiro University. Written informed consent was obtained from each participant.

### 2.2. Materials

In this study, we changed the word order of complex sentences in Japanese, including a quantifier, to manipulate the presence and absence of the syntactic integration of discontinuous constituents, the violation of a syntactic island constraint, and the syntactic prediction in syntactic integration. We constructed sentences of six phrases in Japanese, in which the order of the quantifier and its head noun was changed in a two-by-three way, that is, two precedence relationships between a quantifier and its head noun (Quantifier First and Head-noun First) and three distances between the two (Adjacent, In Syntactic Island, and Distant), as in Table 1.

**Table 1:** Examples of the experimental sentences.

**Phrase 1**	**P2**	**P3**	**P4**	**P5**	**P6**
a. Quantifier First, Adjacent					
Ohta-san-wa	[teien-o	sanpo-shitsutsu]	roku-mai	shashin-o	totta.
Mr/Ms. Ohta-top	[park-acc	a walk-taking]	six	pictures-acc	took.
b. Quantifier First, In Syntactic Island					
Ohta-san-wa	[teien-o	roku-mai	sanpo-shitsutsu]	shashin-o	totta.
c. Quantifier First, Distant					
Ohta-san-wa	roku-mai	[teien-o	sanpo-shitsutsu]	shashin-o	totta.
d. Head-noun First, Adjacent					
Ohta-san-wa	[teien-o	sanpo-shitsutsu]	shashin-o	roku-mai	totta.
e. Head-noun First, In Syntactic Island					
Ohta-san-wa	[teien-o	shashin-o	sanpo-shitsutsu]	roku-mai	totta.
f. Head-noun First, Distant					
Ohta-san-wa	shashin-o	[teien-o	sanpo-shitsutsu]	roku-mai	totta.

*The (possible) propositional meanings of the six sentences were identical to Mr/Ms. Ohta took six pictures (while he/she was) walking in the park. The underlined words indicate quantifiers and their head nouns, and the square brackets ([ ]) indicate adverbial phrases as possible syntactic islands*.

In the current study, we did not assume the a priori effect of a syntactic island violation for Japanese because the syntactic island effect in Japanese could be relatively weak compared to the effect in English. Sprouse et al. (2012) experimentally examined the syntactic island effects in various English constructions. When creating experimental sentences, Sprouse et al. (2012) managed to keep the propositional meaning of a subordinate clause unchanged and manipulated the presence or absence of a construction that could function as a syntactic island. With this manipulation, Sprouse et al. (2012) succeeded in observing the effect of the discontinuous dependency and that of the syntactic island independently. Tokimoto (2019) applied the analysis method in Sprouse et al. (2012) to Japanese sentences that directly corresponded to the experimental sentences in Sprouse et al. (2012), and Tokimoto (2009) demonstrated that the island effect in Japanese was much weaker than that in English. Tokimoto (2009) examined the island effects in Japanese by manipulating the long-distance scrambling of a subordinate object, whereas in the current study, the presence and the absence of a possible island effect were manipulated by a quantifier floating. We were thus careful in predicting the island effect.

The theoretical interests for each word order are summarized below. The dependency relationship between a quantifier and its head noun is expected to be constructed during the fifth phrase (P5). In (c, Quantifier First, Distant) and (f, Head-noun First, Distant) in Table 1, a subordinate clause intervenes between the quantifier and its head noun, and therefore, a syntactic integration process of the discontinuous quantifier and head noun is expected at P5. For the examination of the effect of syntactic integration, (a, Quantifier First, Adjacent) and (d, Head-noun First, Adjacent) are the controls for (c) and (f), respectively. In (c), the following head noun is predicted at the input of a quantifier, whereas in (f), a quantifier is not predicted at the input of a (head) noun.

*Roku-mai* (six) in (b, Quantifier First, In Syntactic Island) and *shashin* (picture) in (e, Head-noun First, In Syntactic Island) have to establish the dependency relationship with *shashin* in (b) and *roku-mai* in (e), respectively. However, one of the two phrases is placed in a possible syntactic island of an adverbial clause in (b) and (e); therefore, the dependency between the two phrases can be difficult to construct.

Furthermore, in (b) and (e), the violation of a syntactic island constraint can be detected at the subordinate verb *sanpo-shitsutsu* (a walk-taking), at which the verb and *teien-o* (park-acc) can construct an adverbial clause. That is, the parser can recognize that a quantifier or a (head) noun is placed in an adverbial clause at the end of the clause even before the parser has received the corresponding head noun or the quantifier. This situation is similar to the example by McKinnon and Osterhout (1996) in that the presence of a *wh*-island can be recognized at *when* placed at the beginning of the subordinate clause.

The quantifiers in the experimental sentences take inanimate nouns as their head nouns. We constructed thirty sentences for each word order: a total of one hundred and eighty sentences. The fifteen subordinate verbs were suffixed by the connective particle *-nagara*, and the others were suffixed by the particle *-tsutsu*, both of which mean *while*. Thirty ungrammatical controls were included in the main session, and in total, two hundred and ten sentences were divided into 3 blocks. The ungrammatical controls were deviant in their argument structures. Two of them are shown in (14).


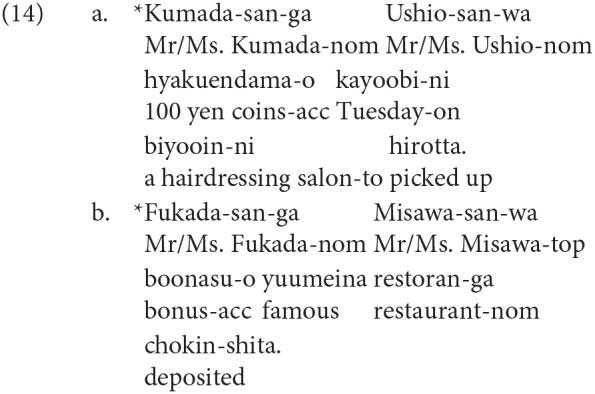


### 2.3. Procedure

The participants were seated in an electrically and acoustically shielded EEG chamber 1 m in front of a 19-inch LCD monitor. A stimulus sentence was visually presented phrase by phrase; the SOA set to 800 ms and the interstimulus interval set to 100 ms. The participants were asked to make grammaticality judgments by a button operation for each sentence (“good” or “bad”). The order of the presentation of the stimulus sentences was randomized for each participant. The experiment was controlled using Presentation (Neurobehavioral Systems). The practice session consisted of ten trials. The main session consisted of three blocks, and the participants were allowed to rest for 3–5 min between the blocks. The experimental sessions, including instruction and the application of the electrodes, lasted approximately 2 h.

### 2.4. EEG Recording

The EEG signals were recorded using a 32-channel EEG amplifier (NuAmps, Neuroscan) with an active electrode recording system (actiCAP, Brain Products; extended 10 – 20 montage). The signals were sampled at 1 kHz with a bandpass filter of 0.1 to 200 Hz with the reference electrode positioned at FCz. Vertical and horizontal electrooculograms (EOGs) were simultaneously recorded from electrodes below the right eye (vertical EOG: VEOG) and at the outer canthi of the left and right eyes (horizontal EOG: HEOGl and HEOGr, respectively). The electrode impedance was maintained at a level lower than 20 kΩ during the sessions. The EEG data were continuously acquired using SCAN (Neuroscan).

### 2.5. EEG Data Preprocessing

The acquired EEG data were processed offline using EEGLAB (Delorme and Makeig, 2004). The preprocessing proceeded as follows. (1) The data were high-pass filtered at 1 Hz to minimize slow drifts. (2) Line noise was removed using the CleanLine plugin in EEGLAB. (3) Artifact subspace reconstruction was used to remove high-amplitude artifacts from the EEG data (Mullen et al., 2015). (4) The EEG data were then re-referenced to a common average reference. (5) The data were decomposed using an adaptive mixture of independent component analyzers (AMICA) (Palmer et al., 2007). (6) The best-fitting single equivalent current dipole was calculated for each independent component (IC) to match the scalp projection of each IC source using a standardized three-shell boundary element head model. The electrode locations corresponding to the extended 10–20 system were aligned with a standard brain model (Montreal Neurological Institute). (7) The possibility of sources for each independent component was evaluated with the ICLabel plugin in EEGLAB (Pion-Tonachini et al., 2017): brain neural activity, EOG, muscle potentials, electrocardiogram, line noise, channel noise, and others. The independent components for which the possibility of brain neural activity was greater than those for the other artifacts were chosen for the following analyses. (8) The data were segmented into time epochs relative to event markers from −1 to 2 s from the markers. (9) The epochs in which the EOG exceeded ±40 μV were rejected.

## 3. Results

### 3.1. Behavioral Data: Grammaticality Judgments

**Figure 1:**
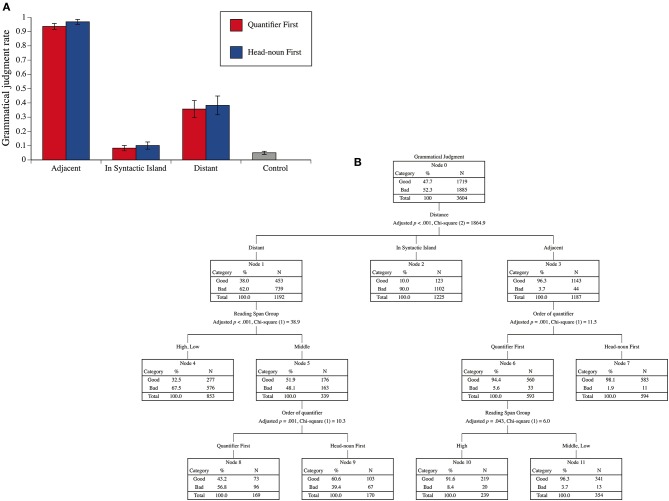
**(A)** Mean “grammaticality” judgment rates of the experimental sentences for the two precedence relationships between a quantifier and its head noun (Quantifier First and Head-noun First) and for the three distances between the two (Adjacent, In Syntactic Island, and Distant) and the mean rate of ungrammatical control sentences. The error bars indicate the standard errors. **(B)** A decision tree for the grammatical judgments with the reading span group, the two precedence relationships between a quantifier and its head noun, the three distances between the two, and the two connective particles at the subordinate verbs (*-tsutsu* and *-nagara*) as independent variables.

The mean proportions of grammaticality judgments for the two precedence relationships between a quantifier and its head noun (Quantifier First and Head-noun First), for the three distances between the two (Adjacent, In Syntactic Island, and Distant), and for the ungrammatical controls are presented in Figure 1A. The participants were divided into three groups depending on their scores of JRST: a high-span group (eight participants with scores greater than the mean score by over 1/2 of the SD), a low-span group (seven participants with scores less than the mean score by over 1/2 of the SD), a middle-span group (the other six participants). Figure 1B shows the decision tree with the grammatical judgments (good or bad) as the dependent variable and with the reading span group, the two precedence relationships between a quantifier and its head noun (Quantifier First and Head-noun First), the three distances between the two (Adjacent, In Syntactic Island, and Distant), and the two connective particles at the subordinate verbs [*-tsutsu* and *-nagara* (while)] as the independent variables. The tree was produced in SPSS version 26 (IBM). The ungrammatical controls were correctly rejected more than 95% of the time. This result suggests that the participants paid enough attention to the task. As Figure 1B indicates, the Adjacent sentences were judged grammatical 96.3% of the time, whereas the mean grammatical judgment rate for In Syntactic Island sentences was 10%. This low rate for In Syntactic Island sentences indicates that the adverbial clauses functioned as syntactic islands. The grammatical judgment rate for Distant sentences was only 38% even though these sentences are generally considered grammatical in the literature of theoretical linguistics. We can recognize a significant effect of the reading span group for Distant sentences in Figure 1B. The participants in the middle-span group accepted more Distant sentences than those in the high- and low-span groups. Some researchers have tried to attribute syntactic island constraints to the constraints of WM (Kluender and Kutas, 1993; Kluender, 1998). As Figure 1B shows, however, the effect of the reading span group was nonsignificant for the judgments of the In Synatic Island sentences. Furthermore, it is not true that a participant with greater WM capacity accepted more Distant sentences, in which a syntactic integration of discontinuous constituents was assumed. It is difficult, therefore, to assume a direct correspondence between the WM capacity and the grammatical judgment of discontinuous dependency as far as our experimental results are concerned. We found no significant effect of the difference of the connective particles at the subordinate verbs on the grammaticality judgments.

### 3.2. ERP Analysis

As the first step for the strict comparison of ERPs, we calculated the mean ERPs elicited by the same words of two conditions for every 50 ms from 100 to 800 ms latency and specified the electrodes at which the difference between the two conditions was determined to be significant by cluster-based permutation tests. We then calculated the mean amplitudes of the electrodes for the two conditions at which the significant difference was observed between the two conditions over 100 ms, and we specified the time window during which the difference between the two mean amplitudes was considered significant by cluster-based permutation tests. The comparison between the two conditions was corrected by cluster-based permutation tests. The analyses of the condition effects in ERP were carried out using the STUDY command structure in EEGLAB. To test the significance of the condition effects, nonparametric random permutation statistics were computed. In the current study, 2,000 random permutations were computed and compared to the *t*- and *F*-values for the mean condition differences.

#### 3.2.1. Syntactic Integration in the Fifth Phrase

To examine the effect of the syntactic integration of discontinuous constituents, we analyzed the ERP time-locked to the onset of the fifth phrase and examined the contrast between Distant and Adjacent of Quantifier First and of Head-noun First sentences.

**Figure 2:**
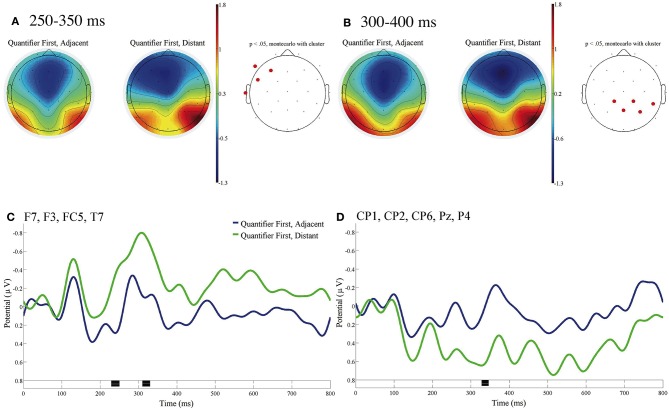
ERPs time-locked to the onsets of the fifth phrases for Quantifier First in **(A–D)** with the baseline from 0 to 100 ms. **(A)** Mean topographies of the ERPs from 250 to 350 ms and **(B)** those from 300 to 400 ms for Adjacent (left) and Distant (right) conditions; the electrode sites at which significant differences were found by using the cluster-based permutation test (*p* < 0.05) are depicted in red (Maris and Oostenveld, 2007). **(C)** Mean ERPs of the two conditions at the left frontal electrodes (F7, F3, FC5, and T7) and **(D)** Mean ERPs of the two conditions at the (right) parietal electrodes (CP1, CP2, CP6, Pz, and P4). Negativity is plotted upward, and the time windows during which significant differences were found by the cluster-based permutation test are indicated in black on the time axis in **(C,D)**.

We analyzed the ERP with the baseline as post-stimulus 100 ms because our intention was to examine the transition of neural activity after the beginning of the syntactic integration process while the word orders up to fourth phrase were different in the Adjacent and Distant sentences. Figure 2 shows the ERP contrasts for the fifth phrases between the Quantifier First sentences with Adjacent and Distant quantifier/head noun relationships [*shashin-o* (picture-acc) in (a) and (c) in Table 1, respectively]. Figure 2A presents the mean topographies of the ERPs from 250 to 350 ms, and Figure 2B presents those from 300 to 400 ms for Adjacent (left) and Distant (right) sentences. Figure 2C shows the mean ERPs of the two conditions at the left frontal electrodes (F7, F3, FC5, and T7), at which significant differences were observed in Figure 2A. Figure 2D shows the mean ERPs of the two conditions at the (right) parietal electrodes (CP1, CP2, CP6, Pz, and P4), at which significant differences were observed in Figure 2B. No significant contrast was observed at the three EOG electrodes (VEOG, HEOGl, and HEOGr) with the time window of 100–1,000 ms.

We found a significant negative ERP in the left frontal region in the time window of 250–350 ms and a significant positive ERP in the (right) parietal region with the time window of 300–400 ms for Distant against Adjacent.

**Figure 3:**
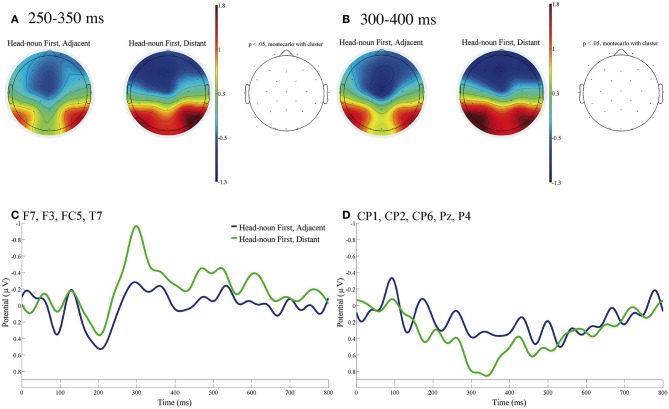
ERPs time-locked to the onsets of the fifth phrases of Head-noun First **(A–D)** with the baseline from 0 to 100 ms. **(A)** Mean topographies of the ERPs from 250 to 350 ms and **(B)** those from 300 to 400 ms for Adjacent (left) and Distant (right) conditions; the electrode sites at which significant differences were found by using the cluster-based permutation test (*p* < 0.05) are depicted in red. **(C)** Mean ERPs of the two conditions at the left frontal electrodes (F7, F3, FC5, and T7) and **(D)** Mean ERPs of the two conditions at the (right) parietal electrodes (CP1, CP2, CP6, Pz, and P4) in the same manner as Figure 2. Negativity is plotted upward, and the time windows during which significant differences were found by the cluster-based permutation test are indicated in black on the time axes in **(C,D)**.

Figure 3 shows the ERP contrasts for the fifth phrases between Adjacent and Distant of Head-noun First sentences [*roku-mai* (six) in (d) and (f) in Table 1, respectively]. Figure 3A presents the mean topographies of ERPs from 250 to 350 ms, and Figure 3B presents those from 300 to 400 ms for Adjacent (left) and Distant (right). Figure 3C shows the mean ERPs of the two conditions at the left frontal electrodes (F7, F3, FC5, and T7), at which significant differences were observed in Figure 2A for Quantifier First sentences. Figure 3D shows the mean ERPs of the two conditions at the (right) parietal electrodes (CP1, CP2, CP6, Pz, and P4), at which significant differences were observed in Figure 2B for Quantifier First sentences again. No significant contrast was observed at the three EOG electrodes in the time window of 100–1,000 ms.

We can recognize the ERP contrast between Adjacent and Distant in Figure 3, which is similar to the contrast in Figure 2. However, the effect in Head-noun First sentences was weaker than that in Quantifier First sentences. We found no electrode at which the contrast between Adjacent and Distant was significant in the time window of 100–1,000 ms.

**Figure 4:**
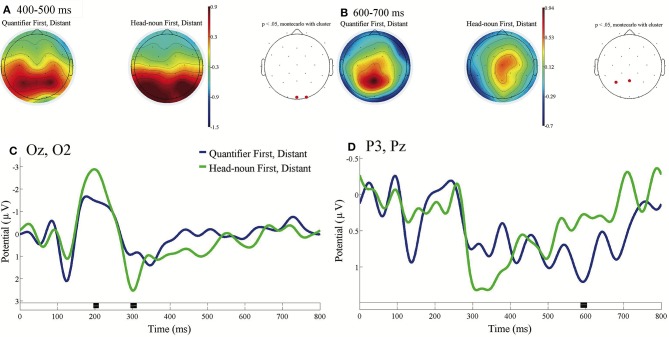
ERPs time-locked to the onsets of the fifth phrases of Distant in Quantifier First and Head-noun First in **(A–D)** with the baseline from 0 to 100 ms. **(A)** Mean topographies of the ERPs from 400 to 500 ms and **(B)** those from 600 to 700 ms for Quantifier First (left) and Head-noun First (right) conditions; the electrode sites at which significant differences were found by using the cluster-based permutation test (*p* < 0.05) are depicted in red. **(C)** Mean ERPs of the two conditions at the occipital electrodes (Oz and O2) and **(D)** mean ERPs of the two conditions at the (left) parietal electrodes (P3 and Pz). Negativity is plotted upward, and the time windows during which significant differences were found by the cluster-based permutation test are indicated in black on the time axes in **(C,D)**.

Figure 4 presents the direct comparison between the ERPs time-locked to the onsets of the fifth phrases of Distant in Quantifier First and in Head-noun First [*shashin-o* in (c) for Quantifier First and *roku-mai* in (d) for Head-noun First in Table 1] with the baseline from 0 to 100 ms. We can observe a significant difference in the occipital region and in the left parietal region.

Steinhauer and Drury (2012) demonstrated that the significance of an ERP deflection could be misjudged depending on the time window of the baseline. We found a significant negativity and positivity for Distant against Adjacent in Quantifier First with the baseline from 0 to 100 ms after the onset of the fifth phrases. However, these significant ERPs could be a spillover effect of a significant deflection in the fourth phrases. We thus analyzed the ERP at the fourth phrases to examine the possible difference in neural activity between Adjacent and Distant conditions that could affect the ERP at the fifth phrases, at which syntactic integration was achieved. We observed a significant positive deflection at the fourth phrases [*roku-mai* (six) for Adjacent (a) in Table 1] and *sanpo-shitsutsu* (a walk-taking) for Distant [(c) in Table 1] in the frontal region for Quantifier First, but the deflection disappeared before the presentation of the fifth phrases. For Head-noun First, we found no significant deflection in the time window of the fourth phrases.

#### 3.2.2. Syntactic Island Violation at the Fifth Phrase

We examined the contrast in ERP between Adjacent and In Syntactic Island conditions at the fifth phrases, at which the discontinuous dependency was expected to be constructed, for either Quantifier First or Head-noun First sentences [*shashin-o* in (a) and (b) for Quantifier First, and *roku-mai* in (d) and (f) for Head-noun First in Table 1, respectively]. However, we found no significant contrast between Adjacent and In Syntactic Island in the time window of 100–1,000 ms after the onset of the fifth phrases with the baseline as 0 to 100 ms latency. We therefore analyzed the ERP contrast at the ends of the adverbial clauses, at which the presence of a syntactic island could be detected.

#### 3.2.3. Syntactic Island Violation at the Subordinate Verb

The violation of a syntactic island constraint can be detected at the subordinate verbs of In Syntactic Island, as we discussed in the materials subsection. Thus, we analyzed the ERP that was time-locked to the onset of the subordinate verbs for In Syntactic Island sentences. In the analysis of the effect of the possible violation of a syntactic island constraint at the end of the adverbial clause, the ERPs time-locked to the onsets of the subordinate verbs [*sanpo-shitsutsu* (a walk-taking)], namely, the fourth phrases of (b) in Table 1 for Quantifier First and of (e) for Head-noun First, were compared to the mean ERP at the subordinate verbs in the third phrases of (a) and (d) as the control because no quantifier or head noun appeared before the subordinate verbs in (a) and (d) and the strings from the first to the third phrases were identical in (a) and (d).

**Figure 5:**
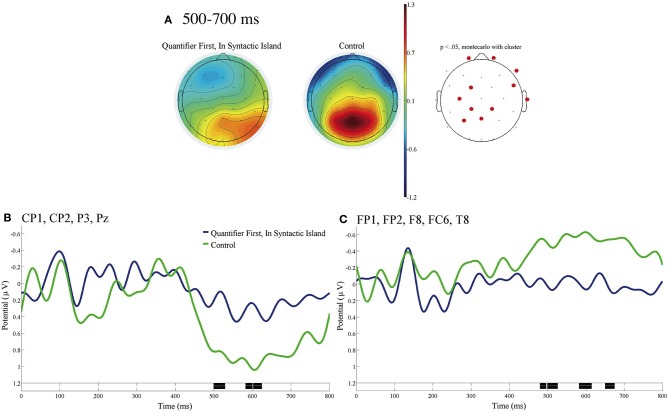
ERPs time-locked to the onsets of the subordinate verbs of Quantifier First and the control in **(A–C)** with the baseline from 0 to 100 ms. **(A)** Mean topographies of the ERPs from 500 to 700 ms for the In Syntactic Island (left) and the control (right) conditions; the electrode sites at which significant differences were found using the cluster-based permutation test (*p* < 0.05) are depicted in red. **(B)** Mean ERPs of the two conditions at the centro-parietal electrodes (CP1, CP2, P3, and Pz), and **(C)** Mean ERPs of the two conditions at the right frontal electrodes (FP1, FP2, F8, FC6, and T8). Negativity is plotted upward, and the time windows during which significant differences were found by the cluster-based permutation test are indicated in black on the time axes in **(B,C)**.

Figure 5 shows the ERP contrast time-locked to the subordinate verbs of In Syntactic Island in Quantifier First sentences and of the control with the baseline from 0 to 100 ms. Figure 5A shows the mean topographies of the ERP from 500 to 700 ms for In a Syntactic Island (left) and the control (right). The electrode sites are depicted in red, where significant differences were found using the cluster-based permutation test (*p* < 0.05). Figure 5B shows the mean ERPs of the two conditions at the centro-parietal electrodes (CP1, CP2, P3, and Pz). Figure 5B shows the mean ERPs of the two conditions at the right frontal electrodes (FP1, FP2, F8, FC6, and T8). No significant contrast was observed at the three EOG electrodes for the time window of 100–1,000 ms. We can recognize a significant negative deflection in the (left) centro-parietal region and a significant positive deflection in the right frontal region for In Syntactic Island against the control.

**Figure 6:**
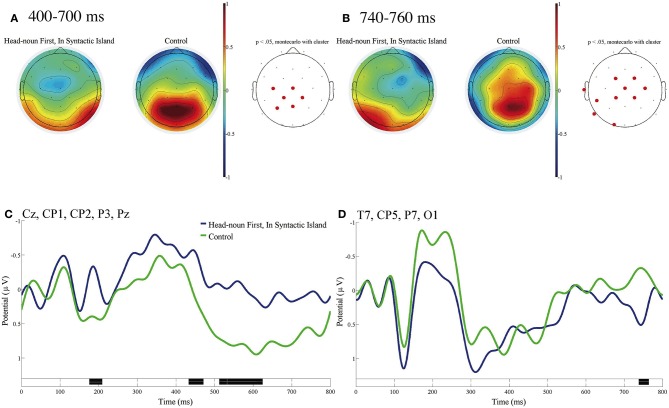
ERPs time-locked to the onsets of the subordinate verbs of In Syntactic Island of Head-noun First and the control in **(A–D)** with the baseline as 0 to 100 ms. **(A)** Mean topographies of the ERPs from 400 to 700 ms and **(B)** those from 740 to 760 ms for the In Syntactic Island (left) and control (right) conditions; the electrode sites at which significant differences were found by using the cluster-based permutation test (*p* < 0.05) are depicted in red. **(C)** Mean ERPs of the two conditions at the (left) centro-parietal electrodes (Cz, CP1, CP2, P3, and Pz) and **(D)** those at the left temporal-occipital electrodes (T7, CP5, P7, and O1). Negativity is plotted upward, and the time windows during which significant differences were found by the cluster-based permutation test are indicated in black on the time axes in **(C,D)**.

Figure 6 shows the ERP contrast that was time-locked to the subordinate verbs of In Syntactic Island of Head-noun First and the control with the baseline from 0 to 100 ms. The mean topographies of ERPs from 400 to 700 ms and from 740 to 760 ms for In Syntactic Island (left) and the control (right) are presented in Figures 6A,B, respectively. The mean ERPs of the two conditions at the five electrodes (Cz, CP1, CP2, P3, Pz) and those at the four electrodes (T7, CP5, P7, O1) are presented in Figures 6C,D, respectively. No significant contrast was observed at the three EOG electrodes with the time window of 100–1,000 ms. We can recognize a significant negative deflection in the (left) centro-parietal region and a significant positive deflection in the left temporal-occipital region for In Syntactic Island against the control condition.

Significant negative ERPs were observed in the centro-parietal region for In Syntactic Island of Quantifier First and of Head-noun First, and the negative ERP was longer in Head-noun First sentences than in Quantifier First sentences. The topographies of the positive ERPs were different in Quantifier First and Head-noun First sentences; that is, the former was observed in the right frontal region, whereas the latter was observed in the left temporal-occipital region. The significant positive ERP was much longer in Quantifier First sentences than in Head-noun First sentences.

We analyzed the ERP at the phrases before the subordinate verbs to examine possible differences in neural activity before the processing of the syntactic island violation. For the Quantifier First condition, the comparison between the ERP at *roku-mai* in (b) and that of *teien-o* (park-acc) in (a) and (d) in Table 1 indicated significant differences in the frontal-central region around the latencies of 500 and 770 ms and in the occipital region around the latency of 600 ms. These significant differences disappeared before the input of the next phrase, namely, the subordinate verb. For the Head-noun First condition, the comparison between the ERP at *shashin-o* (picture-acc) in (e) and that of *teien-o* (park-acc) in (a) and (d) in Table 1 indicated significant differences in the frontal-central region around the latencies of 500 and 770 ms and in the frontal region around the latency of 450 ms. This significant difference also disappeared before the input of the subordinate verb.

**Figure 7:**
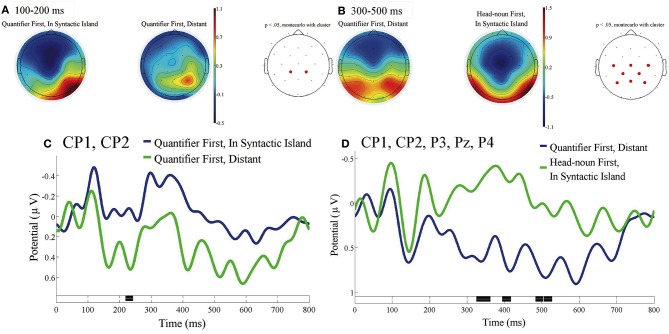
ERPs time-locked to the onsets of the subordinate verbs of In Syntactic Island and of the fifth phrases of Quantifier First, Distant in **(A–D)** with the baseline as 0 to 100 ms. **(A)** Mean topographies of the ERPs from 100 to 200 ms for Quantifier First, In Syntactic Island (left) and Quantifier First, Distant (right), and **(B)** those from 300 to 500 ms for Quantifier First, Distant (left) and Head-noun First, In Syntactic Island (right) conditions; the electrode sites at which significant differences were determined by using the cluster-based permutation test (*p* < 0.05) are depicted in red. **(C)** Mean ERPs of the two conditions at the centro-parietal electrodes (CP1 and CP2) for In Syntactic Island and Distant of Quantifier First and **(D)** those at the centro-parietal electrodes (CP1, CP2, P3, Pz, and P4) for Quantifier First, Distant and Head-noun First, In Syntactic Island. Negativity is plotted upward, and the time windows during which significant differences were determined by the cluster-based permutation test are indicated in black on the time axes in **(C,D)**.

Figure 7 presents the direct comparison between the ERP time-locked to the onsets of the fifth phrases of Distant in Quantifier First [*shashin-o* in (c) in Table 1], the ERP at the subordinate verb of In Syntactic Island in Quantifier First (*sanpo-shitsutsu*) in (b), and the ERP at the subordinate verb of In Syntactic Island in Head-noun First (*sanpo-shitsutsu*) in (e) with the baseline from 0 to 100 ms. We can observe a significant difference in the centro-parietal region between In Syntactic Island and Distant in Quantifier First, and we can observe a significant difference in the centro-parietal broad region between Quantifier First, Distant and Head-noun First, In Syntactic Island in the time window of 330 to 520 ms.

### 3.3. ERSP and ITC Analyses

In this subsection, we will discuss the ERSP and the ITC associated with the syntactic integration and the violation of a syntactic island constraint to examine the neural substrates of the negative ERPs and the positive ERPs in more detail.

#### 3.3.1. ERSP and ITC for Syntactic Integration

**Figure 8:**
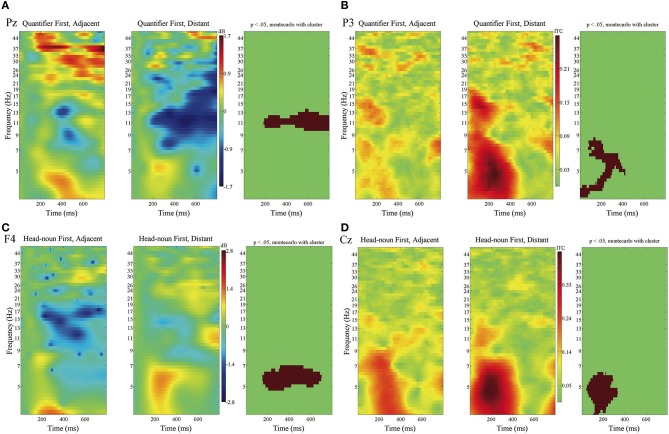
ERSPs and the ITCs time-locked to the onsets of the fifth phrases of Quantifier First and Head-noun First sentences in **(A–D)** with the baseline from 0 to 100 ms. ERSP at Pz in **(A)** and ITC at P3 in **(B)** for Quantifier First, and ERSP at F4 in **(C)** and ITC at Cz in **(D)** for Head-noun First. Time windows and frequency bands are depicted in red, where significant differences were found between Adjacent and Distant by using the cluster-based permutation test (*p* < 0.05).

Figure 8 presents the contrast in the ERSP and the ITC for the fifth phrases between Adjacent and Distant for Quantifier First and Head-noun First for representative electrodes.

For the ERSP for Quantifier First, we can recognize a suppression in the α band for Distant against Adjacent at four electrodes in the centro-frontal region (F8, FC6, CP1, and Pz; Pz in Figure 8A). Conversely, for the ERSP for Head-noun First, we can recognize an enhancement in the θ band at six electrodes in the frontal region (F3, F4, Fz, FC2, C4, and T7; F4 in Figure 8).

The ITC for Quantifier First increased in the δ and θ bands for Distant against Adjacent at seven electrodes in the frontal to occipital regions (FP2, F4, FC5, P3, P7, O1, and Oz; P3 in Figure 8B). Meanwhile, the ITC for Head-noun First increased in the δ and θ bands for Distant at ten electrodes (Fz, F4, FC1, FC2, Cz, P7, P8, O1, Oz, and O2) in the frontal to the occipital regions (Cz in Figure 8D).

#### 3.3.2. ERSP and the ITC for the Violation of the Syntactic Island Constraints

**Figure 9:**
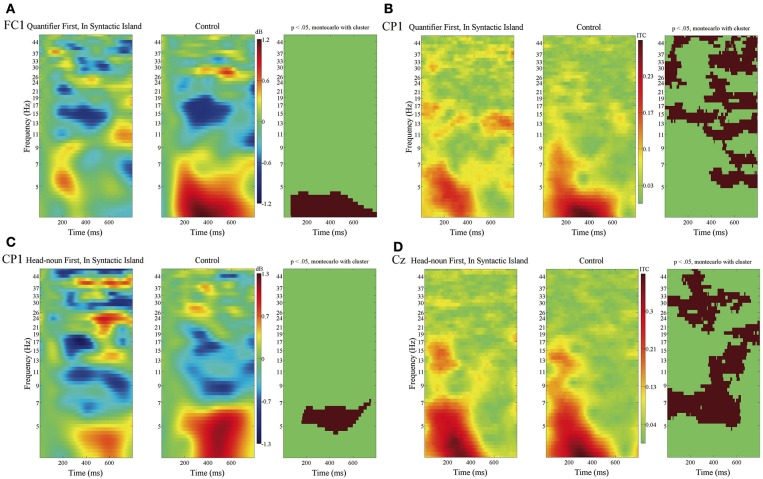
ERSPs and the ITCs time-locked to the onsets of the subordinate verbs of Quantifier First, Head-noun First, and the control condition in **(A–D)** with the baseline from 0 to 100 ms. The ERSP at FC1 in **(A)** and the ITC at CP1 in **(B)** for Quantifier First and the control condition, and the ERSP at CP1 in **(C)** and the ITC at Cz in **(D)** for Head-noun First and the control condition. Time windows and frequency bands are depicted in red, where significant differences were determined between In Syntactic Island and the control condition by using the cluster-based permutation test (*p* < 0.05).

Figure 9 presents the contrast in the ERSP and the ITC between In Syntactic Island and the control condition at the subordinate verbs at representative electrodes.

For In Syntactic Island for Quantifier First, a suppression in the δ band in the ERSP for In Syntactic Island against the control condition was observed at three electrodes in the frontal region (FP1, FP2, and FC1; FC1 in Figure 9A), and the ITC increased in the θ to γ bands at ten electrodes (FP1, FP2, FC1, FC6, C3, CP1, CP2, P3, Pz, and T8; CP1 in Figure 9B). For In Syntactic Island for Head-noun First, a θ suppression was observed at CP1, and the ITC increased in the δ to γ bands in the broad region (C3, CP1, CP2, CP5, Cz, O1, P3, P7, Pz, and T7; Cz in Figure 9D).

### 3.4. Correlations Between Negative ERPs, Positive ERPs, and Japanese Reading Span Test Scores

In this subsection, we will discuss the correlations between the negative ERPs, the positive ERPs, and the individual differences in WM capacity to examine the possible interactions between them, keeping in mind the recent findings that a negative ERP counteracts a positive ERP with respect to semantic anomalies and that the amplitude of the positive ERP elicited by a semantic anomaly was greater for a reader with a greater WM capacity (Kim et al., 2018).

#### 3.4.1. Correlations in Syntactic Integration

Table 2 presents the correlations between the mean amplitude of the negative ERP and that of the positive ERP and between the maximum amplitude of the negative ERP and that of the positive ERP in the time window in which the contrasts between Distant and Adjacent for Quantifier First sentences were significant in Figures 2C,D.

**Table 2:** Means of the mean amplitudes and maximum amplitudes of the ERPs for the fifth phrase in Distant with Quantifier First, with *r* as their correlation coefficients.

	**Negative ERP**	**Positive ERP**	**r**
Electrodes	F7, F3, FC5, T7	CP1, CP2, CP6, Pz, P4	
Time window (ms)	230–330	330–350	
Mean of the mean amplitudes (μV, SD)	−0.080 (0.268)	0.379 (0.817)	−0.648^***^
Mean of the maximum amplitudes (μV, SD)	−1.570 (0.953)	0.856 (1.007)	−0.614^**^

** *p < 0.01*,

*** *p < 0.001*.

The correlations between the negative ERP and the positive ERP were significantly negative for the mean amplitudes and the maximum amplitudes. The amplitudes of the negative ERPs were negative, and thus, the negative correlation coefficients indicate that a reader with a greater negative ERP amplitude showed a greater positive ERP amplitude. Therefore, we cannot find a trade-off between the negative ERP and the positive ERP for syntactic integration; on the contrary, we can recognize that the preceding negativity enhanced the following positivity.

Kim et al. (2018) quantified the N400 and the P600 components for semantically anomalous sentences as mean amplitudes for the time window of 250 to 500 ms and for that of 600 to 850 ms at the central-parietal electrodes (Pz, CPz, Cz, CP1, and CP2) and showed significant correlations between the ERP amplitude and the individual verbal WM capacity. This was because these two time windows were typical for the N400 and the P600, and the central-parietal region was typically maximal for the two ERP components.

There are very few experimental studies on syntactic integration in Japanese, and we found no study that reported an ERP elicited by the violation of a syntactic island constraint in the language. Therefore, it was difficult to predict the latency and the topography for the ERPs elicited by the two linguistic phenomena. We thus analyzed the ERPs and its contrasts in a data-driven approach, as described in the ERP analysis subsection, to specify the latency and the topography.

**Figure 10:**
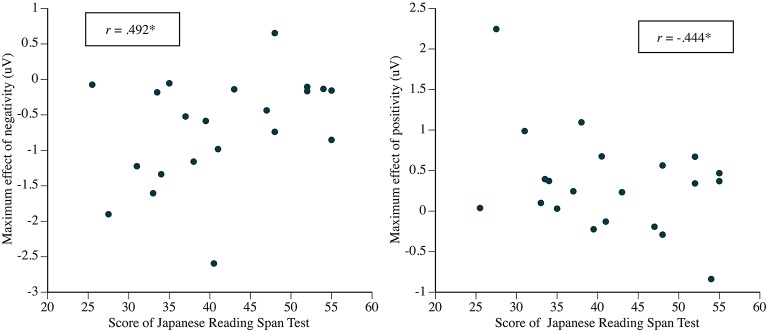
The relationships between the ERP effects and the Japanese Reading Span scores across participants for syntactic integration. Each point represents one participant. The maximum effects are the differences between Distant and Adjacent of Quantifier First in Table 3.

We analyzed the correlation between the ERP amplitude and the score of JRST for the mean/maximum amplitudes at the electrodes and the time windows specified by the data-driven procedure. For the correlation between the effect size of the ERP and the individual WM capacity, we analyzed two indices for the effects: the difference between Distant and Adjacent in the maximum amplitudes of the positive and negative ERPs, and the difference in the mean amplitudes of the two ERPs in the time windows in which the contrast between Distant and Adjacent were significant in Table 2. Table 3 presents the correlations between the two indices of the effect size and the individual score on the JRST, and Figure 10 visually presents the relationships between the ERP effects and the Japanese Reading Span scores across participants for syntactic integration.

**Table 3:** Effect sizes of the ERPs for syntactic integration in Quantifier First and their correlations with Japanese Reading Span Test scores.

**Distant - Adjacent**	**Negative ERPs**	**r**	**Positive ERPs**	**r**
Maximum amplitude (μV, SD)	−0.191 (0.431)	0.492^*^	0.334 (0.795)	−0.444^*^
Mean amplitude (μV, SD)	−0.092 (0.195)	0.271	0.360 (0.629)	−0.406^+^

+ *p < 0.1*,

* *p < 0.05*.

We found a significant positive correlation between the maximum amplitude of the negativity and the JRST score and a significant negative correlation between the maximum amplitude of the positivity and the score. These significant correlations indicate that the effect size of the ERP for syntactic integration is smaller for a reader with a greater WM capacity^3^.

#### 3.4.2. Correlations in a Syntactic Island Violation

Table 4 presents the correlation between the mean amplitude of the negative ERP and that of the positive ERP and the correlation between the maximum amplitude of the negative ERPs and that of the positive ERPs in the time windows in which the contrasts between In Syntactic Island for Quantifier First and the control condition were significant in Figures 5B,C. The correlations between the negative ERPs and the positive ERPs were significantly negative in the mean amplitudes and in the maximum amplitudes. These correlations indicate that a reader with a greater negative ERP showed a greater positive ERP, in the same way as in the syntactic integration part of the experiment. Thus, we do not find a trade-off between the negative ERPs and the positive ERPs in In Syntactic Island for Quantifier First. In contrast, we can recognize a mutual enhancement between the two ERPs.

**Table 4:** Means of the mean amplitudes and the maximum amplitudes for the ERPs at subordinate verbs in In Syntactic Island for Quantifier First.

	**Negative ERPs**	**Positive ERPs**	**r**
Electrodes	CP1, CP2, P3, Pz	FP1, FP2, F8, FC6, T8	
Time window (ms)	500–620	480–670	
Mean of the mean amplitudes (μV, SD)	0.320 (0.636)	−0.061 (0.432)	−0.864^***^
Mean of the maximum amplitudes (μV, SD)	−0.766 (0.804)	0.594 (0.740)	−0.819^***^

*** *p < 0.001*.

**Figure 11:**
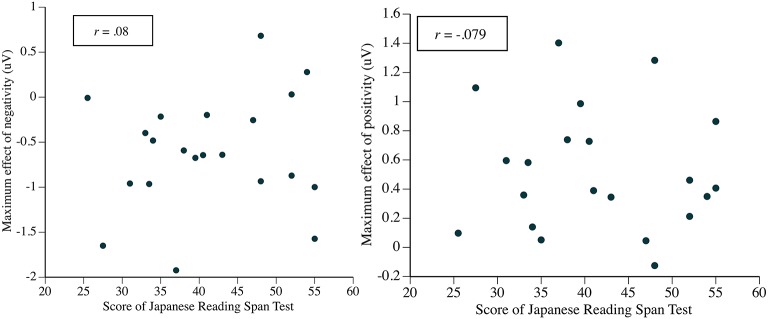
The relationships between the ERP effects and the Japanese Reading Span Test scores across participants for the violation of a syntactic island constraint. Each point represents one participant. The maximum effects are the differences between In Syntactic Island and Control in Table 5.

Table 5 presents the correlations between the two indices of the effect size of the ERPs and the individual scores of JRST for In Syntactic Island for Quantifier First, and Figure 11 presents the relationships between the ERP effects and the JRST scores across participants for the violation of a syntactic island constraint.

**Table 5:** Effect sizes of the ERPs for a syntactic Island violation in In Syntactic Island for Quantifier First and their correlation coefficients with JRST scores.

**In Syntactic Island - Control**	**Negative ERP**	**r**	**Positive ERP**	**r**
Maximum amplitude (μV, SD)	−0.645 (0.605)	0.080	0.524 (0.420)	−0.079
Mean amplitude (μV, SD)	−0.381 (0.411)	0.024	0.337 (0.313)	−0.106

We found no significant correlation between the effect size of the ERP and the JRST score here, in contrast to the syntactic integration part of the experiment.

Table 6 presents the correlations between the mean amplitude of the negative ERPs and that of the positive ERPs and between the maximum amplitude of the negative ERPs and that of the positive ERPs in the time windows in which the contrasts between In Syntactic Island for Head-noun First and the control condition were significant in Figures 6C,D. We find no significant correlation between the amplitudes of the negative ERPs and the positive ERPs.

**Table 6:** Means of the mean amplitudes and the maximum amplitudes for ERPs at subordinate verbs in In Syntactic Island for Head-noun First.

	**Negative ERPs**	**Positive ERPs**	**r**
Electrodes	Cz, CP1, CP2, P3, Pz	T7, CP5, P7, O1	
Time window (ms)	430–630	730–770	
Mean of the mean amplitudes (μV, SD)	0.187 (0.857)	0.212 (0.683)	0.030
Mean of the maximum amplitudes (μV, SD)	−0.948 (0.905)	0.936 (0.881)	−0.148

Table 7 presents the correlations between the two indices of the effect size of the ERPs and the individual scores of JRST for In Syntactic Island for Head-noun First. We found no significant correlation between the effect size of ERP and the JRST score again.

**Table 7:** Effect size of the ERP for a syntactic island violation in In Syntactic Island of Head-noun First and its correlation coefficients with JRST scores.

**In Syntactic Island - Control**	**Negative ERPs**	**r**	**Positive ERPs**	**r**
Maximum amplitude (μV, SD)	−0.380 (0.612)	0.066	0.190 (0.669)	−0.087
Mean amplitude (μV, SD)	−0.291 (0.570)	0.131	0.193 (0.567)	−0.124

Furthermore, we analyzed the correlation between the mean/maximum ERP amplitudes and the grammaticality judgment rates for Distant and In Syntactic Island conditions. We found no significant correlation between the judgment rates and the mean/maximum amplitudes for the negativity and the positivity for the syntactic integration and for the syntactic island violation. Furthermore, we found no significant correlation between the grammaticality judgment rates and the JRST scores^4^.

## 4. Discussion

The first research question of the current study is to differentiate between the ERPs, especially the late positive ERPs, elicited by a syntactic integration and the violation of syntactic island constraints in the same construction with the same lexical items. The significant ERPs associated with syntactic integration (when a quantifier preceded its head noun) were the left frontal negative ERP with the time window of 250–350 ms and the parietal positive ERP with the time window from 300 to 400 ms. On the other hand, the significant ERPs associated with the violation of a syntactic island constraint were the parietal negative ERP and the right frontal positive ERP in the time window of 500–700 ms when a quantifier was placed in the syntactic island. When the head noun was placed in the syntactic island, a parietal negative ERP with the time window of 400–700 ms and a left temporal-occipital positive ERP were significant. Furthermore, the direct comparison between the ERP at the fifth phrases of Quantifier First, Distant and the ERPs at the subordinate verbs in Quantifier First and Head-noun First of In Syntactic Island indicated significant contrasts between the syntactic integration and the syntactic island violation (Figure 7). Thus, we believe it is reasonable to claim that the ERP associated with the syntactic integration and that associated with the violation of syntactic island constraints were different in their latencies and topographies.

We will discuss the theoretical implications of our result to the cross-linguistic understanding of the ERP below. Yano et al. (2019) is one of the recent studies that reported the P600 for a syntactic violation in Japanese. Yano et al. (2019) constructed Japanese sentences consisting of one noun case-marked by a postposition and a verb, in which they changed the combination of case-makers and the transitivity of a verb to manipulate the grammaticality of a sentence. Some experimental sentences in Yano et al. (2019) are given in (15) and (16). The subject in (16-a) is phonetically null, but (16-a) is grammatical if the subject is understood as the speaker. Yano et al. (2019) observed the P600 for (b) against (a) in (15) and (16).


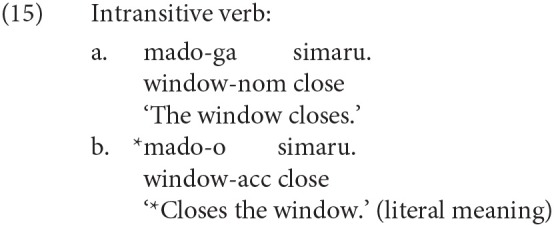



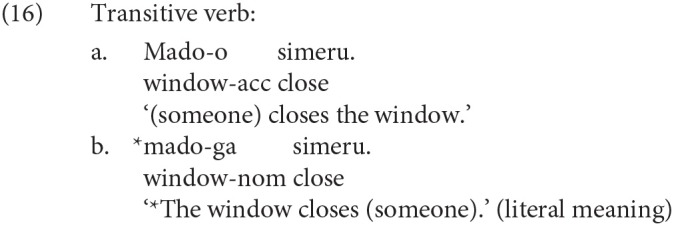


The cause of the ungrammaticality of (15-b) and (16-b) is the agreement error between the subject/object and the verb, whereas the syntactic violation examined in this study is the violation of a syntactic island constraint. To the best of our knowledge, the current study is the first to report a late positive ERP for the violation of a syntactic island constraint in Japanese.

For the syntactic island violation in this study, a broad negative ERP was observed in addition to a positive ERP, and the topography was different from the topography of the late positive ERP for a syntactic island violation in English. Further research is necessary to explain why a syntactic island violation in Japanese is strongly associated with a negative ERP. Here, we will note the possibility that the differences in word order between English and Japanese due to their head directions could be related to the salient negative ERP for Japanese. In the example of syntactic island violation in McKinnon and Osterhout (1996) reproduced here as (17-a), a positive ERP was observed for *when* at the beginning of the subordinate clause. As is schematically shown in (17-b), a *wh*-phrase *which of his staff members* precedes the corresponding word (*by*), and it turns out that at *when*, the corresponding word is in a syntactic island.


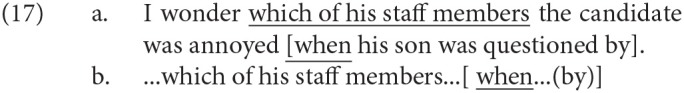


We can assume here that the input of the corresponding word is predicted at *which of his staff members* with its lexical information kept in WM, and it turns out that at *when*, the prediction will not be satisfied because the corresponding word is placed in a syntactic island.

The type of a clause can be judged at the beginning of the clause in English due to its head-first nature, whereas it cannot be determined in Japanese until the end of the clause because Japanese is head-final. In processing the sentences of In Syntactic Island in (18-a,b), therefore, it turns out that at the subordinate verb *sanpo-shitsutu* (a walk-taking), a quantifier *roku-mai* (six) in (18-a) and the head noun *shashin* (picture) in (18-b) are in a syntactic island. Here, the detection of a syntactic island depends on the building of a verb phrase with *teien-o* (park-acc) and *sanpo-shitsutu* with their semantically natural relationship and on the judgment that *roku-mai* and *shashin-o* (picture-acc) cannot be a clause mate of the subordinate verb *sanpo-shitsutu*.


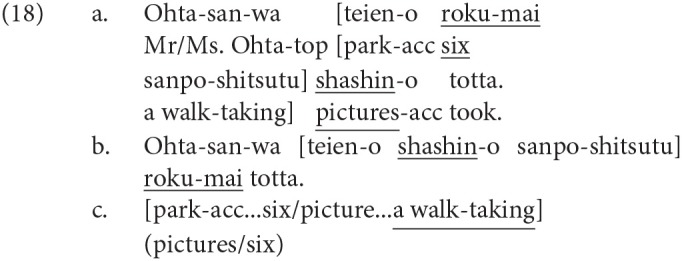


In (17-a), the presence of a syntactic island can be detected by one word: *when*. In (18-a,b) in Japanese, on the other hand, the judgment and the construction of the appropriate dependency relationship between a subordinate verb and its object is necessary for the detection of a syntactic island and its violation. Therefore, the detection of a syntactic island violation in Japanese can be more related to semantic processing than that in English.

It is true that the processes that followed the syntactic island violation in Japanese could involve semantic processing but note that the syntactic violation in (18) is caused only by changing the word order. The anomaly in (18) is thus a syntactic phenomenon. On the other hand, the examples of Kim et al. (2018), which are reproduced below, cannot be well-formed by changing their word order.


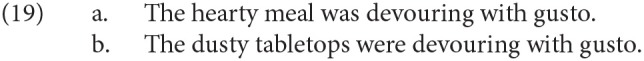


We can thus understand the anomaly of (19) to be semantic.

We believe that the positive ERP for (18-a) longer than that for (18-b) is suggestive here. That is, in (18-a), the head noun is predicted at the input of a quantifier (six), and it turns out that at the subordinate verb *sanpo-shitsutu*, the prediction will not be realized. In (18-b), on the other hand, it turns out that at the subordinate verb, *shashin-o* (picture-acc) is in a syntactic island, but the following quantifier is not predicted here, in contrast to (18-a). If one of the causes of a late positive ERP is an unsatisfied syntactic prediction, the unsatisfied prediction can be a property common to the positive ERP for (17) in English and that for (18-a) in Japanese. It is thus possible that the ERP topographies for syntactic island violations in English and Japanese are different because of the interaction between the different processes for the detection of a syntactic island and its violation due to the different word order, the possible involvement of semantic processing, and the presence or absence of a syntactic prediction.

We can interpret the left anterior negativity for the syntactic integration in the time window of 250–350 ms (as in Figure 2) in the current study as a manifestation of the additional processing load on WM. King and Kutas (1995) examined the ERP in the processing of relative clauses in English as in (20), and they observed a left anterior negativity (LAN) during the relative clause and *admitted* in the object relative against the subject relative.


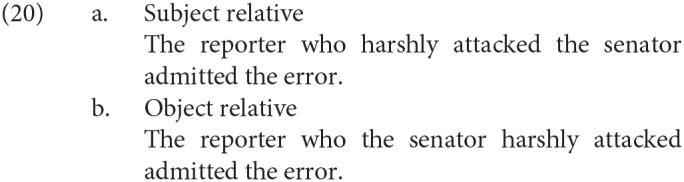


King and Kutas (1995) interpreted the LAN in (20-b) as the manifestation of the additional load on the WM and the discharge of it. That is, in the object relative, the filler (*the reporter*) is retained during the processing of the relative clause and is retrieved at the input of *attacked* to construct the dependency between the verb (*attacked*) and its object (*the reporter*). In the subject relative, on the other hand, the parser encounters *attacked* earlier than in the object relative, and therefore, the load on the working memory is smaller in the subject relative than in the object relative. In Distant conditions in the current study, one of the discontinuous words could be retained during the processing of the adverbial clause, and the dependency relation would be constructed at the other word at the fifth phrase. Therefore, the negativity in the left anterior region would appear in Quantifier First, Distant condition.

We also found significant contrasts in ERSP between Quantifier First and Head-noun First for the syntactic integration. That is, the integration in Quantifier First was characterized by a suppression in the α band, whereas that in Head-noun First was characterized by an enhancement in the θ band. As for the ITC for syntactic integration and syntactic island violation, we found an increase in the θ band with the time window of 100–400 ms for the former (Figure 8B) and an increase in the θ to γ bands for more than 800 ms after the onset of the subordinate verb for the latter (Figures 9B,D). We can thus assume that the neural activity for syntactic island violations was more induced than that for syntactic integration.

The second research question of this study is the possible counteraction between the negative ERP and the positive ERP when the ERPs are biphasic. The left frontal negative ERP and the parietal positive ERP for the syntactic integration in Quantifier First were negatively correlated in the mean and the maximum amplitudes (as in Table 2). These correlations indicate that a reader with a greater negative ERP showed a greater positive ERP. We thus find no counteraction between the negative ERP and the positive ERP in the syntactic integration. As for the syntactic island violation, the negative ERP and the positive ERP at the subordinate verb in Quantifier First were negatively correlated in the mean and the maximum amplitudes (as in Table 4). Here again, a reader with a greater negative ERP showed a greater positive ERP for the syntactic island violation. Therefore, we cannot recognize a counteraction between the negative ERP and the positive ERP that was reported for the ERP elicited by semantic anomalies. To the best of our knowledge, the current study is the first to find positive correlations between the amplitude of the negative ERP and that of the positive ERP in the syntactic integration and the syntactic island violation in Japanese. The interactive enhancement of the negative ERP and the positive ERP can be characteristics of syntactic processing, although further experiments should be performed to examine the possible interaction of the ERPs associated with semantic anomalies in Japanese.

The third research question of this study is to examine the effect of syntactic prediction on syntactic integration. As Figures 2, 3 show, the contrast between Distant and Adjacent was significant when a quantifier preceded its head noun, whereas the contrast did not reach a significant level when the head noun preceded the quantifier. This finding indicates that the syntactic prediction was deeply associated with the ERP associated with syntactic integration. To the best of our knowledge, the current study is the first to present evidence that syntactic prediction can enhance the ERP for the construction of discontinuous dependency. Furthermore, as Figures 8A,C shows, an α suppression was observed when the quantifier preceded the head noun, whereas a θ enhancement was observed when the head noun preceded the quantifier. These suppressions and enhancements suggest that the α band is concerned with the retention of the lexical information of a quantifier in WM and its clearance at syntactic integration and that the θ band is associated with the retrieval or the backtracking of the head noun at the quantifier. As for the effect of the syntactic prediction for the head noun by a quantifier on the detection of a syntactic island violation, we also found a significant contrast in the ERSP for In Syntactic Island as in Figures 9A,C. That is, we recognized a δ suppression in Quantifier First and a θ suppression in Head-noun First. This difference in the frequency band could be a manifestation of the presence or absence of a syntactic prediction, but the number of electrodes was fewer, at which the contrast was significant, than that in syntactic integration. The effect of syntactic prediction was thus smaller in a syntactic island violation than in syntactic prediction.

The fourth research question of this study is the possible correlation between the ERP amplitude and WM capacity. As shown in Table 2, the amplitude of the negative ERP and that of the positive ERP for syntactic integration (in Quantifier First) were significantly correlated with the individual differences in the JRST scores. As we briefly reviewed above, Kim et al. (2018) found that a reader with a greater WM capacity showed a smaller negative ERP and a greater positive ERP for semantic anomalies. In the current study, on the other hand, a reader with a greater WM capacity showed a smaller negative ERP and a smaller positive ERP. Kim et al. (2018) and the current study both claim a systemic relationship between the neural activity for sentence processing and WM capacity. However, the current study contrasts with Kim et al. (2018) in the correlation between the amplitude of the positive ERP and the individual capacity of WM. It will be straightforward to assume that the amplitude of the ERP for syntactic integration is a manifestation of its processing load relative to the individual capacity of WM because the lexical information of a quantifier will be retained in WM in Distant for Quantifier First and the retention will be cleared at the syntactic integration. Thus, the amplitude of the ERP for a reader with a greater WM capacity is smaller than that for a reader with a smaller WM capacity. We can thus predict no correlation between the amplitude of the ERP for a syntactic island violation and the JRST score because the retention process of the distant phrase is absent in a syntactic island violation. We believe that our result is the first evidence to show a systematic relationship between the neural activity for syntactic integration and WM capacity and to show no relationship between the activity for a syntactic island violation and WM capacity. According to Kim et al. (2018), a structural reanalysis requires the verbal WM to temporarily retain the sentence representation in the mind while seeking a plausible interpretation, and individuals with greater WM capacity are more likely to initiate structural reprocessing in response to an unexpected word because they have greater WM capacity for the reanalysis. In contrast, individuals with a smaller WM capacity are assumed to avoid structural reanalysis and respond to an unexpected word by attempting to retrieve a meaning appropriate to the context. When we accept the claim by Kim et al. (2018), WM constrains a possible reanalysis and the retrieval of an appropriate meaning in a semantically anomalous sentence, whereas the WM functions to retain the lexical information of the preceding word and to clear the syntactic prediction at the construction of the discontinuous dependency in syntactic integration. We can understand that the difference in the correlations between the amplitude of the ERP and WM capacity between Kim et al. (2018) and the current study was a manifestation of the different roles of WM in semantic anomaly and syntactic integration.

## 5. Limitations

The amplitudes of the significant ERPs observed in the current study were relatively small compared to those in many other previous studies, as one of the anonymous reviewers pointed out. We believe that we succeeded in the artifact rejection to detect a small difference in ERP. However, the exact reason for the small ERP is unknown.

We interpreted the left centro-parietal effect for Quantifier First, In Syntactic Island to be a negative deflection in comparison to the control condition. We assumed that the ERP at the subordinate verbs of (a) and (d) in Table 1 should be appropriate as the control because no quantifier or head noun was included in the string from the first to the third phrases in (a) and (d). However, it is possible in principle that the negative ERP for In Syntactic Island was actually the positive deflection of the control.

Nieuwland and Van Berkum (2006) examined the correlation between the ERP elicited by the resolution of an ambiguous pronoun in the context and the individual difference of WM capacity by a split-group analysis to show that the neural activity was qualitatively different between a reader with a small WM capacity and a reader with a great capacity. We attempted a split-group analysis for our ERPs with the participants divided into three groups, namely, high-, middle-, and low-span groups. However, we failed to find a significant difference between the high-span group and the low-span group. The reason for the absence of the qualitative difference can be the relatively small number of participants (twenty-one) for the split-group analysis.

The underlying processes of negative and positive ERPs (N400 and P600) is a large issue under intense discussion (Gouvea et al., 2010; Brouwer et al., 2012; Brouwer and Crocker, 2017). As one of the proposals, van de Meerendonk et al. (2008) proposed the monitoring hypothesis for the N400 and the P600, as we briefly discussed in the introduction section. According to van de van de Meerendonk et al. (2008), the P600 reflects a reanalysis at a strong conflict between expectancies and what is observed, whereas the N400 is the manifestation of a successful reanalysis and the integration at a conflict. We believe cross-linguistic studies will be helpful for a deeper understanding of linguistic ERPs and the neural mechanisms of sentence processing in general.

## 6. Conclusion

Discontinuous dependency is universal in natural languages, and thus, syntactic integration can occur in any language. A syntactic island is also universal, though the types of constituents that construct it vary among languages (Goodluck and Rochemont, 1992). However, the information needed to detect the linguistic phenomena and their processing order can be different among languages. Furthermore, because the manner of the involvement of WM can vary depending on the processing order, the real-time neural activity for a universal phenomenon can be different among languages.

One of the main points of the current study is that linguistic processing can be associated with multiple ERP components and that one component can bear different theoretical meanings depending on the relationship with the other component(s) and the individual difference of the participants. We should emphasize the positive correlation in the ERP amplitudes between the negativity and positivity associated with the syntactic integration and with the syntactic island violation, whereas the correlation between the negativity and positivity elicited by the semantic anomaly reported by Kim et al. (2018) was negative. This positive correlation can be characteristic of syntactic processing. We should be cautious when assuming a direct correspondence between an ERP component and a linguistic phenomenon.

## Data Availability Statement

The datasets generated for this study are available on request to the corresponding author.

## Ethics Statement

The studies involving human participants were reviewed and approved by the ethics committee of Mejiro University. The patients/participants provided their written informed consent to participate in this study.

## Author Contributions

ST: design of the work, acquisition of data, analysis, interpretation of data, and manuscript preparation. YM: making of the materials. NT: acquisition of data, analysis, and interpretation of data.

### Conflict of Interest

The authors declare that the research was conducted in the absence of any commercial or financial relationships that could be construed as a potential conflict of interest.

## Footnotes

^1^*Mai* is a numeral classifier generally used for papery objects.

^2^We can find some studies that examined the effect of a syntactic prediction on ERP (Lau et al., 2006 in English and Jiang and Zhou, 2009 in Chinese).

^3^We analyzed the correlation between the ERP amplitudes and the JRST scores for the Head-noun First condition, although we found no significant difference between Distant and Adjacent of Head-noun First. We found a significant negative correlation between the maximum effect of the negativity in the left frontal region and the JRST score at the electrodes and the time window examined for Quantifier First (*r* = 0.536^*^). We found no significant correlation between the parietal positivity and the JRST score (*r* = −0.223 for the maximum effect).

^4^Tokimoto (2009) examined the correlation between the effect of the syntactic island in Japanese and the individual capacity of WM by a self-paced reading experiment on Japanese complex sentences involving discontinuous dependency manipulated by long-distance scrambling. Tokimoto (2009) observed significant island effects for the long-distance scrambling from a complex noun phrase and from an adverbial clause. However, Tokimoto (2009) found no significant correlation between the grammaticality judgment rates for the syntactic islands and the JRST scores. Tokimoto (2009) claimed that the syntactic island phenomenon in Japanese was independent of the WM constraints, which was consistent with Sprouse et al. (2012), who examined the relevance of WM capacity to the syntactic island in English.
